# Gender Differences in Parental Impact on Problematic Smartphone Use among Korean Adolescents

**DOI:** 10.3390/ijerph18020443

**Published:** 2021-01-08

**Authors:** Hyunmi Son, Suwon Park, Gyumin Han

**Affiliations:** 1 College of Nursing, Pusan National University, Yangsan 50612, Korea; hangyum@pusan.ac.kr; 2 College of Nursing and Health Innovation, University of Texas at Arlington, Arlington, TX 76019, USA; suwon.park@uta.edu

**Keywords:** adolescent, behavior, addictive, parents, parent-child relations, smartphone

## Abstract

This study aimed to examine the effective parental impact in preventing problematic smartphone use in adolescents by identifying the parent-related factors. A secondary analysis of cross-sectional data from a Korean national survey was conducted. Data from 2758 male and 2419 female adolescents, aged 10 to 19 years, were analyzed; the respondents were divided into normal and risk groups based on their standardized smartphone addiction diagnostic scale scores. Parent-related factors of smartphone addiction were analyzed using a logistic regression model. Among both male and female adolescents, mobile messenger usage, and family environment emerged as significant predictors of problematic smartphone use. In addition, for male adolescents, smartphone use frequency and parent’s awareness of content use were significant predictors. The findings indicated the importance of parental roles in preventing problematic smartphone use in adolescents. Parents should create a healthy family environment by avoiding smartphone overuse and modeling the appropriate usage of smartphones.

## 1. Introduction

Smartphone use has significantly increased with the development of its features and applications since the advent of smartphones in the early 2000s. According to statistics that surveyed the global smartphone penetration rate in 2019, it was found that 44.9% of the world’s population owns a smartphone [1], and 93.6% in South Korea [2]. Using a smartphone, users can communicate, search for information, connect to social networks, play games, download music or videos, read books, take photos, record videos, and shop online. Although the development of smartphones brings many benefits to people’s lives, smartphone use has negative effects, the most concerning being smartphone addiction. Uncontrolled or excessive smartphone use leads to many social, psychological, and health problems [3]. Problematic smartphone use, referring to the behavioral disorder, which is considered a repetition and continuation of a behavior despite its adverse effects on one’s life [4], has gained public attention.

For all these multitasks available in a small portable device, the majority of the population, especially adolescents enjoy smartphone use, and the amount of time and frequency in use of smartphones have remarkably increased [5]. According to a study on smartphone addiction among adolescents and adults, 9.2% of adults were in the smartphone addiction or addiction risk group, and adolescents were 18.6% because adolescents tend to be less self-controlled to the urge to pursue pleasure [6]. Among Korean adolescents, 30.3% report being overdependent on smartphones [7], which is higher than the reports of smartphone addiction in other countries [8,9,10]. Excessive smartphone use among adolescents can interfere with concentration at school or work and can cause physical difficulties, such as neck stiffness, blurred vision, wrist or back pain, and sleep disturbances [11,12,13,14]. It can also reduce in-person social interaction and academic achievement and lead to mental health problems [11,14,15]. Therefore, smartphone overuse is a major problematic behavior issue to consider in adolescents.

According to Mcleroy’s social-ecological theory [16], adolescents experience interactions with their surroundings, such as parents, teachers, siblings, and peers. Among them, the parental impact is very important in adolescents’ problematic behavior [17]. Parents strive to help their children grow in the right direction in adolescents’ problematic behavior. However, the basis for how important parents influence on the children’s use of smartphones is insufficient.

Behaviorism viewed it as a learned behavior that is subject to the stimulus-response-reinforcement principle [18]. Thus, like any other learned behavior, smartphone addiction can be modified [19]. Adolescents under parental regulation and supervision are reported to have a decreased risk of misconduct [19,20]. Parents manage their children’s use of smartphones by limiting the amount of time they use, limiting the number of messages that can be sent, or limiting the content of smartphone use [21]. However, some researchers, regarding smartphone overuse, have stated that parental regulation and interference, negatively perceived by adolescents, elevate children’s problematic behaviors [22,23]. Studies need to identify the kind of method that is effective for parents to discipline their children’s problematic smartphone usage.

In addition, according to the attachment theory, secure attachments with parents provide warmth and support to children [24]. Many studies showed that the poorer parent-child relationship is related to the more problematic behavior [17]. A positive parent-child relationship reduces adolescent problematic behaviors [25]. In the relationship, perceived parental trust plays a key role in reducing problematic behaviors [26]. However, few studies have been conducted on the effects of parent-child relationships on children’s smartphone use.

Research on adolescent behavior has suggested that parental behavior is influential [17], and specifically, imitation of negative behaviors have been demonstrated within parent-child relationships [27]. Thus, this research holds value for those in parental roles in considering their potential influence on their children. Moreover, parental behavior creates a family environment that directly affects the child’s experience [28]. In an environment where parents spend a great deal of time on media with their children, children would learn to use such media [29]. Therefore, a parent’s smartphone habit can affect their children’s smartphone use.

The factors influencing adolescents’ smartphone addiction need to be identified by dividing men and women. There is a difference in substance abuse among male and female adolescents due to previous experiences and psychological differences [30]. Male and female adolescents show differences in smartphone usage patterns [31]. Male adolescents mainly use smartphones for entertainment, such as games, whereas female adolescents use smartphones for communication with other people [31]. Moreover, depending on gender in the parent-child relationship, there are differences in nurturance, closeness, discipline, and authoritativeness [32]. Therefore, the study aimed to investigate the influencing factors on problematic smartphone use focusing on parent-related variables that have a major impact on adolescents in male and female adolescents.

## 2. Methods

### 2.1. Design and Participants

The study analyzed secondary data obtained from a Korean national survey on internet addiction [33]. This national survey is conducted annually; however, only the survey in 2014 included data on parent-related factors. Thus, we utilized the data obtained from the 2014 survey data that utilized a cross-sectional design and was conducted from September through November in 2014. A total of 18,500 participants reported their use of smartphones in the survey. The participants were people aged 3 to 59 years who have used the internet and smartphone at least once within a month from 1 September 2014. Stratified random sampling was used to obtain a representative sample in the survey. The extraction units were geographically selected across 17 provinces and cities using systematic sampling. For the present study, data of 2758 male and 2491 female adolescents (10 to 19 years old) were analyzed.

### 2.2. Variables and Measures

#### 2.2.1. Problematic Smartphone Use

The standardized smartphone addiction diagnostic scale (S-scale) developed by the National Information Society Agency (NIA) of South Korea was used to measure the risk of smartphone addiction [34]. This scale has 15 items in the following four domains: (1) disturbance of adaptive functions, (2) virtual life orientation, (3) tolerance, and (4) withdrawal. It is a self-administered questionnaire in which responses to each item are scored on a four-point scale, with 1 indicating “never” and 4, “always.” The following three groups are classified based on the sum of scores and 1, 3, and 4 domain scores: normal, potential risk, and high-risk users. In the present study, the potential and high-risk users were referred to as problematic smartphone users. Based on this, problematic smartphone users were designated as the risk group and normal users as the normal group.

#### 2.2.2. Demographics and Characteristics of Smartphone Use

The variables measured were gender, age, parental economic activity, frequency of daily smartphone usage, duration of smartphone usage, frequency of daily mobile messenger usage, use of unlimited data, and use of parental control software.

#### 2.2.3. Factors Related to Parents

##### Parental Discipline

To measure parental discipline on the use of smartphones, we used the questions asked whether parents take the actions of each item on the use of smartphones by adolescents. There were five items, we corded “My parents know how much I use my smartphone” as ‘knowing the duration of usage,’ “My parents know what I’m using on my smartphone” as ‘knowing the content browsed on the smartphone,’ “My parents and I have rules for using smartphones” as ‘having rules,’ “My parents introduce me to useful applications or teach me how to find information through my smartphone” as ‘informing how to use a smartphone,’ and “My parents and I play smartphone games together or communicate via messenger” as ‘sharing interests’. Each item was rated on a five-point Likert scale ranging from 1 = “strongly disagree” to 5 = “strongly agree”.

##### Parent-Child Relationship

We used the following six questions to measure the parent-child relationship; “My parents understand me,” “My parents have faith in me,” “When I have trouble, I tend to discuss with my parents,” “My parents tend to listen to my opinion,” “My parents are very interested in me,” and “My parents are good at praising me.” Each question was recorded as “parental understanding,” “parents’ belief,” “parents’ discussion of concerns,” “parents’ listening to opinion,” “parental interest in the child,” and “parental praise.” Each item was measured on a 5-point Likert scale.

##### Family Environment

As a question to measure the family environment, an open-ended question “How many members of the family are using the smartphone excessively?” was used. Those who answered more than one person were classified as “existence of family members who overuse”, and none were classified as “non-existence of family members who overuse.”

### 2.3. Data Analysis

The data were analyzed using SPSS 23.0 (IBM, Armonk, NY, USA), with statistical significance set at *p* < 0.05. The data were weighted for the statistical adjustment prior to analysis [33]. Frequency, percentage, mean, and standard deviation were used to describe the participants and their characteristics of smartphone use. The χ^2^-test and *t*-test were used to identify differences in characteristics between the at-risk and normal groups. Logistic regression analysis was performed to determine the factors associated with smartphone addiction in male and female adolescents.

### 2.4. Ethical Consideration

Prior to conducting the research, the researchers received the approval of the institution review board of their university (PNU IRB/2016_77_HR). Statistical data was used with approval from NIA. The researchers of this study utilized data, except personal identification information, after participant anonymity and confidentiality were guaranteed. The analysis was performed in accordance with the NIA’s statistical analysis recommendations.

## 3. Results

### 3.1. Demographics and Prevalence of Smartphone Addiction

As shown in Table 1, the mean age of the participants was 15.1 (SD = 2.58) years. The age of the participants was 10–13 years old, 30%, 14–16 years old 35.2%, 17–19 years old 34.8%. The ratio by age was similar among men and women. More than half of the participants had dual-income parents, and 28.6% of male adolescents and 29.9% of female adolescents were in the smartphone addiction risk group.

### 3.2. Differences in Demographics, Characteristics of Smartphone Usage, and Factors Related to Parents between the Two Groups

Table 2 presents the differences between normal and risk groups for male and female adolescents. Among both male and female adolescents, variables showed significant differences between the two groups, namely, the frequency of daily smartphone (*p* < 0.001) and daily mobile messenger usage (*p* < 0.001). Regarding parental discipline over smartphone use for male adolescents, the normal group showed higher scores compared with the at-risk group in knowing the duration of usage (*p* = 0.049) and knowing the content use (*p* < 0.001). Unlike the male adolescents, the female adolescents in the normal group showed higher scores compared with the at-risk group in having rules (*p* = 0.013) and informing them how to use them (*p* = 0.010). Regarding the parent-child relationship for male adolescents, parental understanding (*p* < 0.001), parents’ belief (*p* = 0.005), parents’ discussion of concerns (*p* = 0.012), parents’ listening to opinion (*p* = 0.017), and parental praise (*p* = 0.036) were statistically significant. Among female adolescents, parental understanding (*p* = 0.029), parents’ belief (*p* = 0.013), parents’ discussion of concerns (*p* < 0.001), parents’ listening to opinion (*p* = < 0.001), and parental praise (*p* = 0.013) showed significant differences between the two groups. For both male and female adolescents, there was no statistically significant parental interest in the child. The rate of family members who overused smartphones were significantly higher in the at-risk group than in the normal group for male (*p* < 0.001) and female adolescents (*p* < 0.001).

### 3.3. Factors of Problematic Smartphone Use among Male and Female Adolescents

The factors influencing the problematic smartphone use of male adolescents are presented in Table 3, and the results for female adolescents are in Table 4. Among male adolescents, the represented model fit the data well (Hosmer–Lemeshow χ^2^ = 4.819 [df = 8]; *p* = 0.777), and was statistically significant (χ^2^ = 88.189, *p* < 0.001). Among female adolescents, the model also fit the data well (Hosmer–Lemeshow χ^2^ = 4.864 [df = 8]; *p* = 0.772), and was statistically significant (χ^2^ = 103.033, *p* < 0.001). The higher the frequency of use of smartphones for male adolescents, the higher the risk of becoming a smartphone problem user by 1.013 times (*p* = 0.008). The higher the frequency of using mobile messengers, the higher the probability of smartphone use problems was 1.015 times (*p* = 0.001). Among female adolescents, the frequency of daily mobile messenger usage and family environment emerged as significant predictors of smartphone addiction. When parents regulate the content of smartphone use, male adolescents have a 0.797 times (*p* = 0.039) lower risk of overusing smartphones. In particular, it was found that male adolescents have a 2.005 times (*p* < 0.001) higher risk of overusing smartphones if there are members of the family that overuse smartphones. For female adolescents, frequency of daily mobile messenger usage (OR = 1.026, *p* < 0.001) and existence of family members who overuse smartphones (OR = 1.855, *p* < 0.001) were significant predictors.

## 4. Discussion

We investigated parent-related factors in preventing problematic smartphone use in male and female adolescents. The analysis focused on the factors related to parents and family environment in smartphone use for male and female adolescents. Among our participants, 28.6% of male adolescents and 29.9% of female adolescents were categorized in the risk group for smartphone addiction. Compared with adolescent smartphone addiction rates in other countries, such as Switzerland (16.9%) [9], the UK (13.3%) [10], and Lebanon (22.1%) [8], the smartphone addiction rate among Korean adolescents is much higher and has been increasing [7]. The smartphone penetration rate of South Korea is the highest in the world, at 95% [2], which might have contributed to the increase in adolescent smartphone overuse and could continue to affect smartphone overuse in even younger children [23]. Indeed, adolescent smartphone overuse and overdependence will inevitably continue to increase globally. Therefore, the results of this study, using Korean national data, are expected to provide important evidence for future research.

When exploring family or parental influences, we found that the family environment of smartphone use is the most influential factor in smartphone addiction among all other variables in both male and female adolescents. Male adolescents have a 2.005 times higher risk of becoming a problematic smartphone user if there are excessive smartphone users in their families, and female adolescents 1.855 times higher. This finding is similar to that in a previous study in which parents’ screen time is strongly associated with children’s screen time [29]. The family environment may have a greater impact on adolescent behaviors than formal learning because children learn through integrating cognitive and emotional perceptions in the environment where they are placed [35]. Adolescents who observe problematic behavior in their families are at high risk of imitating their behavior [27]. This has been proven in a study showing that adolescents’ alcohol use, binge drinking, and marijuana use were affected by parental substance abuse [36]. Moreover, parents who are overly immersed in the media tend to be tolerant of their children’s use of media devices [37], and are less likely to respond to the needs of their children while using the device [38], which may lead to problematic behaviors of adolescents. Therefore, parents should be aware of the impact of their smartphone use on their children’s behaviors. Building a healthy and safe family environment for smartphone use can be the first step to preventing adolescent smartphone addiction.

In addition, the frequency of daily mobile messenger usage was an influencing factor in both male and female adolescents. Adolescents use smartphones more for relationship purposes; that is, to build and maintain their relationships [3,39]. They are engaged in social networking, using smartphones to build and maintain relationships through communication with friends and others, and their attachment to smartphones can lead to addiction. The relationship-oriented nature of smartphone use in adolescents should be considered when developing addiction prevention programs. In addition, institutional regulatory measures should be formulated to prevent excessive use of social media networks as well as mobile messengers among adolescents. For example, users can limit the usage time and set the alarm, knowing the endpoint through a push notification sent by the applications [40]. However, the function of the application does not shut the application down, so the users can continue to stay on it. Therefore, we cannot guarantee that this application is effective for those who have low self-discipline, such as adolescents. We should consider simple but stronger ways to prevent smartphone overuse in female adolescents.

Influencing factors that differed between male and female adolescents were the frequency of daily smartphone use and knowing content use in parental discipline. The higher the frequency of use of smartphones among male adolescents, the higher the risk of using problematic smartphones was 1.015 times, and the risk was 0.797 times lower when parents regulated the content of smartphone use. However, female adolescents were not affected by both variables. The difference between male and female adolescents can be explained by the results of previous studies that showed the risk of addiction differing according to the smartphone usage patterns of adolescents. Male adolescents use their smartphones primarily to play games and watch videos. Female adolescents use smartphones as a means of communication [31]. These results suggest that parents should consider gender differences when disciplining their children with respect to smartphone use.

In this study, variables relating to parent-child relationship were not shown to be effective in reducing the risk of smartphone addiction in male and female adolescents. This is inconsistent with the research showing that adolescents have a low risk of becoming addicted to smartphones if the relationship between parents and children is positive [25,41]. In this study, it is interpreted that these results were obtained by analyzing factors affecting adolescents’ smartphone considering the interaction of various parent-related variables. Therefore, to examine closely the effect of parent-child relationships, we suggest repetitive further research to investigate the influencing factors of parent-related variables.

Although this study provided significant findings, it has several limitations. For one, the use of a cross-sectional design meant that could not determine causal relations. In addition, to include the variables related to parents, it was unavoidably analyzed using data performed in 2014. However, looking at the smartphone penetration rate in Korea, it is already about 80% in 2014 [2], which exceeded the global average in 2019, and this could be generalized and applied. Moreover, the psychological factors of adolescents, such as self-esteem or depression, which affect interpersonal relationships, were not considered in the study. However, we examined parent-related factors in various aspects. Given the lack of research on parent-child relationships, our findings can be used to provide a guide on how parents should manage their children’s smartphone use for preventing problematic smartphone use. Public health providers could use these results in parental education and family intervention in preventing children’s problematic smartphone usage.

## 5. Conclusions

This study investigated parental discipline, parent-child relationship, and family environment, which are variables related to parents, to identify the factors influencing the problematic use of smartphones for male and female adolescents in South Korea. As a result, both male and female adolescents were affected by the frequency of daily mobile messenger usage and the existence of family members who overuse smartphone. Additionally, male adolescents were found to be affected by the frequency of daily smartphone usage and parental discipline of smartphone usage contents. These results could contribute to the development of interventions for the increasing public concern over the smartphone addiction of adolescents.

Based on the results of this study, parents should create a family environment so that adolescents do not become problematic smartphone users. Parents can shape their family environment by avoiding overuse and using smartphones appropriately in front of their children. Therefore, public health providers should educate parents so that they can construct an effective family environment to prevent adolescents from using problematic smartphones. When applying adolescents’ smartphone-related interventions, it is necessary to develop an intervention that reflects their characteristics in consideration of gender. In particular, it is necessary for male adolescents to strictly manage their usage time and regulate what they use with smartphones.

## Figures and Tables

**Table 1:** Demographics and prevalence of smartphone addiction (*n* = 5249).

Characteristics	Total	Male (*n* = 2758)	Female (*n* = 2491)
	Unweighted Frequency (Weighted %)	Unweighted Frequency (Weighted %)	Unweighted Frequency (Weighted %)
Age (M ± SD)	15.11 ± 2.53	15.13 ± 2.48	15.10 ± 2.58
10–13 years (Grade 3–6)	1541 (30.0)	789 (29.1)	752 (30.9)
14–16 years (Grade 7–9)	1884 (35.2)	1016 (36.4)	868 (33.9)
17–19 years (Grade 10–12)	1824 (34.8)	953 (34.5)	871 (35.2)
Parental economic activity			
Dual-earners	3189 (59.2)	1662 (58.5)	1527 (59.9)
Single-earner	2060 (40.8)	1096 (41.5)	964 (40.1)
Smartphone user type			
Normal group	3786 (70.8)	2019 (71.4)	1767 (70.1)
Risk group	1463 (29.2)	739 (28.6)	724 (29.9)

**Table 2:** Differences in demographics, characteristics of smartphone usage, and factors related to parents between the two groups (*n* = 5249).

Categories	Male (*n* = 2758)			Female (*n* = 2491)		
	Normal	Risk	χ^2^/t (*p*)	Normal	Risk	χ^2^/t (*p*)
	*n* (%)/M ± SD	*n* (%)/M ± SD		*n* (%)/M ± SD	*n* (%)/M ± SD	
Age(years)						
10~13	580 (71.0)	209 (29.0)	0.747 (0.688)	543 (71.7)	209 (28.3)	4.713 (0.095)
14~16	735 (70.5)	281 (29.5)		579 (66.1)	289 (33.9)	
17~19	704 (72.9)	249 (27.1)		645 (72.6)	226 (27.4)	
Parental economic activity						
Dual-earners	1195 (69.8)	467 (30.2)	2.157 (0.142)	1083 (70.0)	444 (30.0)	0.007 (0.933)
Singer-earner	824 (73.7)	272 (26.3)		684 (70.3)	280 (29.7)	
Frequency of daily smartphone usage	20.59 ± 12.30	26.34 ± 24.87	−4.173 (<0.001)	20.88 ± 12.16	28.68 ± 27.01	−5.150 (<0.001)
Duration per use (minute)	13.54 ± 11.59	14.13 ± 14.37	−0.701 (0.484)	13.25 ± 11.92	12.53 ± 12.84	0.884 (0.519)
Frequency of daily mobile messenger usage	12.26 ± 16.19	20.09 ± 25.94	−5.258 (<0.001)	12.20 ± 12.50	20.94 ± 24.00	−6.380 (<0.001)
Use of unlimited data plan						
Yes	239 (67.2)	121 (32.8)	1.949 (0.163)	195 (65.6)	101 (34.4)	1.195 (0.166)
No	1736 (72.1)	608 (27.9)		1554 (71.0)	616 (29.0)	
Use of parental control software						
Yes	642 (72.5)	216 (27.5)	0.374 (0.541)	579 (72.5)	220 (27.5)	0.481 (0.488)
No	1376 (70.9)	523 (29.1)		1188 (70.2)	504 (29.8)	
Parental discipline of smartphone use						
Knowing duration of usage	2.55 ± 0.662	2.47 ± 0.676	−1.974 (0.049)	2.56 ± 0.685	2.54 ± 0.647	0.459 (0.646)
Knowing content use	2.49 ± 0.740	2.33 ± 0.706	3.609 (<0.001)	2.49 ± 0.725	2.41 ± 0.712	1.669 (0.095)
Having rules	2.57 ± 0.745	2.52 ± 0.707	1.207 (0.228)	2.56 ± 0.768	2.44 ± 0.719	2.490 (0.013)
Informing how to use	2.31 ± 0.683	2.23 ± 0.669	1.885 (0.060)	2.33 ± 0.703	2.22 ± 0.681	2.586 (0.010)
Sharing interests	2.59 ± 0.713	2.54 ± 0.744	1.238 (0.216)	2.61 ± 0.695	2.54 ± 0.721	1.580 (0.114)
Parent-child relationship						
Parental understanding	2.85 ± 0.551	2.74 ± 0.584	3.036 (0.002)	2.92 ± 0.542	2.83 ± 0.600	2.186 (0.029)
Parents’ belief	3.06 ± 0.637	2.95 ± 0.618	2.838 (0.005)	3.06 ± 0.629	2.96 ± 0.679	2.489 (0.013)
Parents’ discussion of concerns	2.85 ± 0.719	2.74 ± 0.692	2.517 (0.012)	2.97 ± 0.678	2.81 ± 0.706	3.694 (<0.001)
Parents’ listening to opinion	2.92 ± 0.644	2.82 ± 0.656	2.393 (0.017)	2.98 ± 0.643	2.81 ± 0.671	3.888 (<0.001)
Parental interest in child	3.01 ± 0.656	2.94 ± 0.663	1.625 (0.104)	3.02 ± 0.671	2.94 ± 0.666	1.818 (0.069)
Parental praise	2.79 ± 0.631	2.71 ± 0.628	2.095 (0.036)	2.82 ± 0.608	2.72 ± 0.662	2.484 (0.013)
Family environment						
Existence of family members who overuse	812 (65.1)	442 (34.9)	27.293 (<0.001)	688 (63.9)	419 (36.1)	21.367 (<0.001)
Non-existence of family members who overuse	1207 (78.5)	297 (21.5)		1079 (76.3)	305 (23.7)	

Note: *n* is unweighted and % is weighted.

**Table 3:** Factors of problematic smartphone use among male adolescents (*n* = 2758).

Characteristics	Categories	OR (95%CI)	*p*
Characteristics of smartphone use	Frequency of daily smartphone usage	1.013 (1.003–1.022)	0.008
	Frequency of daily mobile messenger usage	1.015 (1.006–1.024)	0.001
Parental discipline	Knowing duration of usage	1.071 (0.846–1.357)	0.568
	Knowing content use	0.797 (0.643–0.989)	0.039
Parent-child relationship	Parental understanding	0.859 (0.647–1.141)	0.294
	Parents’ belief	0.858 (0.677–1.087)	0.203
	Parents’ discussion of concerns	0.966 (0.770–1.211)	0.763
	Parents’ listening to opinion	1.031 (0.798–1.332)	0.818
	Parental praise	0.984 (0.751–1.289)	0.905
Family environment	Existence of family members who overuse smartphone (reference: no)	2.005 (1.526–2.635)	<0.001

Note. OR = odds ratio; CI = confidence interval.

**Table 4:** Factors of problematic smartphone use among female adolescents (*n* = 2491).

Characteristics	Categories	OR (95% CI)	*p*
Characteristics of smartphone use	Frequency of daily smartphone usage	1.007 (0.997–1.018)	0.161
	Frequency of daily mobile messenger usage	1.026 (1.014–1.038)	<0.001
Parental discipline	Having rules	0.917 (0.759–1.109)	0.374
	Informing how to use	0.898 (0.729–1.105)	0.309
Parent-child relationship	Parental understanding	1.111 (0.821–1.502)	0.495
	Parents’ belief	0.927 (0.734–1.170)	0.523
	Parents’ discussion of concerns	0.906 (0.717–1.145)	0.409
	Parents’ listening to opinion	0.770 (0.590–1.005)	0.054
	Parental praise	0.972 (0.735–1.287)	0.845
Family environment	Existence of family members who overuse smartphone (reference: no)	1.855 (1.410–2.442)	<0.001

Note. OR = odds ratio; CI = confidence interval.

## Data Availability

The data used in this study can be used by requesting data disclosure from the National Information Society Agency in South Korea.
